# A modified Park's technique for creating a natural-looking double eyelid

**DOI:** 10.4314/ahs.v23i2.55

**Published:** 2023-06

**Authors:** Sichun Zhao, Chong Zou, Tailing Wang, Jiaqi Wang

**Affiliations:** Facial and Cervical Plastic and Cosmetic Surgery Center, Chinese Academy of Medical Sciences & Peking Union Medical College Plastic Surgery Hospital and Institute, Beijing, China

**Keywords:** Blepharoplasty, Levator aponeurosis, Orbicularis oculi, Dermis, Fixation

## Abstract

**Objective:**

This study aimed to evaluate an improved park's technique to create a natural double eyelid and reduce scar formation.

**Methods:**

The levator aponeurosis, orbicularis oculi muscle, and dermis were fixed using subcutaneous intermittent sutures, and then the skin and the levator aponeurosis were sutured. Postoperative evaluation included double-eyelid stability, symmetry, double-eyelid curve, scar formation, and patient satisfaction.

**Results:**

Between January 2021 and August 2021, a total of 89 patients (178 eyes) underwent double-eyelid blepharoplasty by the same surgeon using this improved technique. The mean follow-up period was 15.5 months (range 10 to 18 months). Seventy-six patients were very satisfied with this surgical method, six patients were rather satisfied, and seven patients were dissatisfied. The overall satisfaction rate was 92.1%.

**Conclusion:**

This new blepharoplasty method generates a stable, natural double-eyelid line with minimal scarring.

## Introduction

Since Mikamo first described double-eyelid surgery in 1896, various surgical methods have been proposed to construct the double eyelid^1^. As blepharoplasty achieves a more vivid eye shape that is considered esthetically pleasing, this procedure has remained the most popular cosmetic surgery in China for years^2^.

Although the mechanism of double-eyelid formation has not been fully elucidated, the main surgical principle is to connect the aponeurosis or tarsus with the pretarsal skin^3^. At present, double-eyelid blepharoplasty is mainly divided into the incisional method and the buried suture method^4^. The incisional method is the most commonly used blepharoplasty procedure in China because it creates a stable double eyelid. However, the traditional incisional method of creating a double eyelid involves extensive resection of the orbicularis oculi muscle and the anterior tarsal fascia, which can result in unnatural folds and hypertrophic scars; other common complications are ecchymosis, edema, and a long recovery time^5^.

In 1999, Park proposed a surgical method to fix the orbicularis oculi muscle and the levator aponeurosis to form a double-eyelid crease^6^. In his theory, this approach is both effective and stable. Meanwhile, Park's method has a shorter recovery period than the traditional method. However, in our clinical experience, this kind of adhesion is somewhat unsteady and recurrence is a common problem. Even Park mentioned the frequent relapse as a common complication without a good solution^7^.

To obtain a more natural and vivid double-eyelid fold with a quick recovery time and minimal complications, we improved the incisional technique of double-eyelid blepharoplasty. The improved procedure creates an upper eyelid structure that is similar to the anatomy of a congenital double eyelid and can be widely used in clinical practice.

## Materials and Methods

This study was approved by the institutional review board of the Peking Union Medical College and Plastic Surgery Hospital & Institute (approval No. YLMR00821134), and performed according to the tenets of the Declaration of Helsinki.

### Patients

Between January 2021 and August 2021, a consecutive series of 89 patients (178 eyes) who had undergone double-eyelid blepharoplasty using this improved technique were included in this retrospective observational study. The cohort comprised 83 women and six men with a mean age of 26.8 years (range 18–37 years). Patients who had undergone previous upper eyelid surgeries or combined with blepharoptosis were excluded from the study. All operations were performed by the senior author (Chong Z) with the patients under local anesthesia. The patients provided consent for the analysis of their personal information, operative procedures, and outcomes.

### Preoperative Evaluation

In preoperative evaluation, the palpebral crease line was designed based on each patient's desired appearance. The patient was seated while the doctor gently pressed the skin of the upper eyelid with a fine probe and asked the patient to close their eyes naturally to determine the expected crease height. The patient was then asked to open their eyes, look in the mirror, and check whether they were satisfied with the height of the double-eyelid crease. In general, the preferred height of the crease above the lashes was 5–7 mm for men and 6–8 mm for women.

### Surgical Technique

All surgeries were performed under local anesthesia with an injection of 2% lidocaine containing 1:100,000 epinephrine. The skin of the upper eyelid was cut, and a 2–5-mm-wide strip of the orbicularis oculi muscle was removed in accordance with the skin redundancy. The upper eyelid was tweezed at a predetermined crease until the skin was taut and the lashes began to move; this gave a good estimate of the amount of excess skin. The orbicularis oculi muscle under the incision was carefully removed with tissue scissors, and bipolar electrocoagulation was used for accurate hemostasis. The removal of the orbicularis oculi clearly revealed the orbital septum. When there was too much orbital fat, the orbital septum was cut laterally and the appropriate amount of orbital fat was removed based on the degree of eyelid swelling. Care was taken to avoid eyelid depression and thoroughly stop the bleeding.

**Figure 1:**
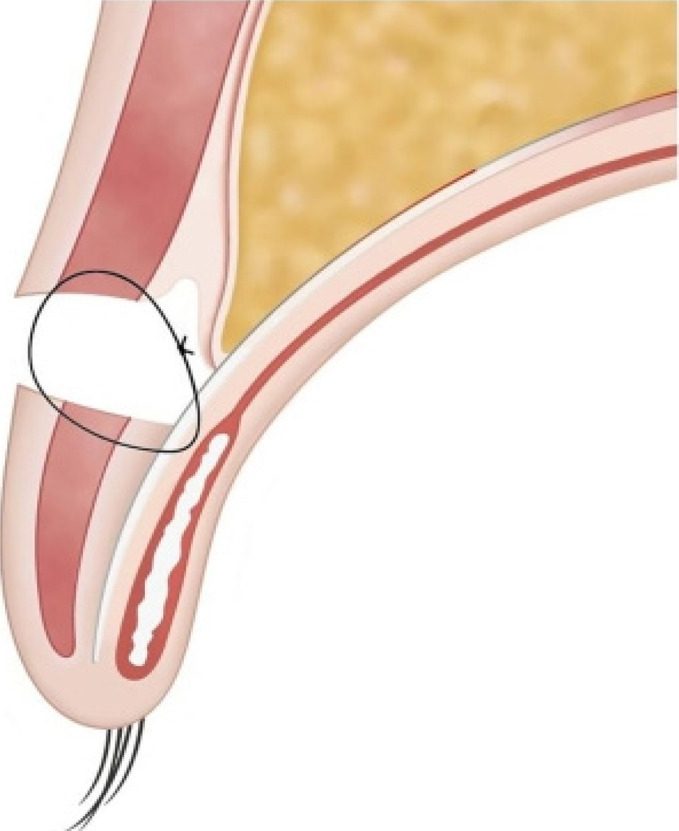
Diagram of the improved double-eyelid surgical technique

The levator aponeurosis, orbicularis oculi muscle at the lower edge of the incision and its dermis, and orbicularis oculi muscle at the upper edge of the incision were sutured successively with 7-0 monofilament suture (Figure 1). Care was taken to deeply bury the suture knots. The position of the orbicularis oculi muscle-dermis complex fixed to the aponeurosis of the upper eyelid determined the contour of the double eyelid, usually at the level of the upper edge of the tarsus. The levator muscle fascia and skin were then sutured with 7-0 monofilament interrupted sutures. During the operation, patients were asked to open their eyes and observe the shape, height, and symmetry of the double eyelids, which were adjusted to the patient's satisfaction. Immediately after surgery, the eyelid was cooled with an ice bag for 2 hours. The patient was then discharged and instructed to apply an ice bag as much as possible for 2 days postoperatively. External sutures were removed 7 days after surgery. In addition, patients were advised to avoid applying makeup on the upper eyelid for 7 days after suture removal.

### Outcome Assessment

Subjective and objective methods were used to assess the surgical results. For subjective assessment, patient satisfaction was evaluated by a self-assessment questionnaire after a 10-month follow-up period. Patient satisfaction with the surgical outcome was classified as follows: “very much,” “rather,” and “not at all” satisfied. Objective assessment was performed by 3 independent observers: 2 plastic surgeons and a profile photographer. Postoperative evaluation included double-eyelid stability, symmetry, double-eyelid curve, and scar formation. The observers were asked to provide a score of 1 to 6 for each the 4 specific above-mentioned areas.

## Results

**Figure 2:**
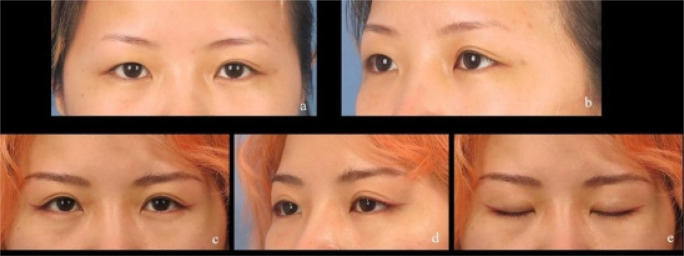
**a, b:** Preoperative photographs of a 26-year-old Chinese woman. c–e: Photographs taken at 10 months postoperatively show a beautiful upper eyelid curve without obvious scarring

**Figure 3:**
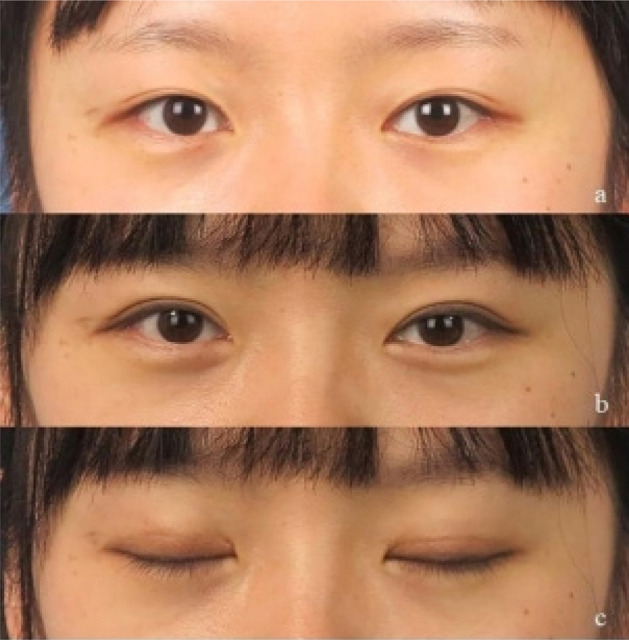
**a:** Preoperative photographs of a 23-year-old woman. b, c: Photographs taken at 9 months postoperatively show no visible scarring when the eyes are closed

This novel approach was performed successfully in all patients. The patients were followed up for 10 months to 18 months (mean, 15.5 months). Most patients showed satisfactory cosmetic results. There were 76 patients (85.3%) who were very much satisfied with this surgical method, six patients (6.7%) who were rather satisfied, and seven (7.8%) who were not at all satisfied. The overall satisfaction rate was 92.1% (Figure 2 and 3). No serious complications such as corneal injury occurred.

Postoperative recovery was uneventful. Six patients developed bruising but the tissue recovered within 1 week. Four eyes showed somewhat unsmooth folds. Most patients had slight upper eyelid swelling, but recovered within 2-3 weeks. The average scores of the shape of double-eyelid by outside observers also demonstrated good surgical outcome for the stability (5.3±0.4), symmetry (5.0±0.8), and Eyelid curve (5.2±0.5) than for the scar formation (4.8±0.7).

## Discussion

According to Sayoc's levator expansion theory, the anatomical basis of the natural double eyelid is that the fibers of the levator fascia extend to the anterior dermis of the tarsus distally through the orbicularis oculi muscle^8^. When the eyes are open, the levator palpebrae superioris contracts and pulls up the tarsus; this pulls up the skin of the upper eyelid, forming a fold. The congenital single eyelid occurs due to: (1) a lack of fibers of the levator muscle reaching the skin or fusion of the levator muscle and the orbital septum below the upper edge of the tarsus; (2) thick skin and subcutaneous tissue hindering the formation of folds9. Therefore, the aim of blepharoplasty is to establish a connection between the skin and the levator muscle or tarsus10, remove excess upper eyelid tissue, and establish a firm adhesion between the levator muscle or tarsus and the skin.

Traditional blepharoplasty is performed by suturing the skin to the tarsus. In the classic double-eyelid method proposed by Sayoc in 1954, the orbicularis oculi muscle in front of the tarsus is removed, and the skin is sutured to the tarsal plate to create a firm scar adhesion to form a stable double eyelid^11^. Although the direct suturing of the tarsus and skin in this method can form the double eyelid in a stable manner, the created folds are usually static and unnatural because the tarsus is a dense connective tissue without sliding characteristics. The removal of the orbicularis oculi muscle in front of the palpebra not only leads to an obvious step, but also often leads to obvious scar hyperplasia. In addition, it takes a long time to reconstruct the arteriovenous and lymphatic reflux around the eye after surgery, resulting in swelling for 6 months or longe^12^.

To obtain a natural and stable double eyelid, many scholars have modified the abovementioned method. These modifications include creating the double eyelid by fixing the orbital septal flap with skin^13^, suturing the white line of the levator aponeurosis to the orbicularis oculi muscle^14^, suturing the orbicularis oculi muscle and tarsus plate^15^, internally suturing the orbicularis muscle, aponeurosis, and tarsus, using the aponeurosis of the upper eyelid as the connecting tissue to directly transfer the force to the skin^16^,^17^, and total resection double-eyelid blepharoplasty with selective neurovascular preservation^12^. Although these methods are better than the traditional double-eyelid technique, they still do not mimic the anatomy of the congenital double eyelid.

Park proposed the orbicularis oculi-levator aponeurosis fixation technique in 1999. In Park's method, the orbicularis oculi muscle and the aponeurosis of the levator muscle of the upper eyelid are sutured and fixed to form the upper eyelid fold^6^. Because of its rapid recovery and natural effect, Park's method has been widely used in China. However, in Park's method, the adhesion is not solid enough and recurrence is a common problem^18^. The reason for recurrence may be that the loosening of the fixed line or the repeated pulling of the aponeurosis of the upper eyelid leads to relaxation of the aponeurosis, and the resultant failure of the aponeurosis to conduct effective power transmission leads to the disappearance of the double eyelid line. Furthermore, although Park's method involves suturing of the aponeurosis of the levator and the orbicularis oculi muscle so that the eye-opening force is transmitted to the orbicularis oculi muscle, it does not directly transmit to the dermis and fails to imitate the anatomy of the double eyelid in the natural state.

Lu et al.^16^ proposed the use of the levator aponeurosis of the upper eyelid to construct a physiologically natural double eyelid. The levator aponeurosis of the upper eyelid was used as the “connecting tissue” to transfer the power of the levator muscle to the upper eyelid and suture it with the skin in a planar way, thus achieving a natural double-eyelid effect. Both the method of Lu et al. and our method emphasize the conduction of power from the aponeurosis to the skin and obtain natural and stable surgical results without removing the orbicularis oculi muscle in front of the tarsus. However, the method of Lu et al. involves extensive dissection of the aponeurosis above the tarsus. In our opinion, extensive dissection of the aponeurosis is undesirable because it increases the possibility of aponeurosis injury and prolongs the swelling time.

In our method, the levator aponeurosis and the incision of the upper orbicularis oculi muscle and the dermis are sutured and fixed, which more similar to the natural double eyelid regarding the anatomical structure and the force transmission during eye opening. The aponeurosis of the upper eyelid is directly fixed to the orbicularis oculi muscle and dermis, obtaining natural and long-lasting surgical results. Our method is suitable for most Asian patients who want natural-looking double eyelids.

The orbicularis oculi muscle at the upper and lower edges of the incision was sutured with the levator aponeurosis, simulating the complete annular orbicularis oculi muscle structure of the normal eyelid. This markedly reduced the skin tension of the incision, reduced incisional scar hyperplasia, effectively shortening the postoperative recovery time. In the present series of patients, the swelling was completely resolved in only 2–3 weeks, and the incision of the upper eyelid appeared smooth without steps or unevenness with the eye closed. The curve of the normal upper eyelid was retained, which meets the esthetic requirements. To stabilize the double-eyelid line, the aponeurosis of the levator and skin were sutured again when the incision was closed to ensure that the strength of the levator muscle of the upper eyelid could be transmitted to the skin. The double suture and fixation effectively avoided single-eyelid fold recurrence and obtained a natural, stable, and lasting double-eyelid fold.

There were no significant complications in the present case series. Four eyes showed some unsmooth folds, which might be caused by loose sutures or a suture foreign body reaction leading to the formation of a local subcutaneous cyst. In most of these patients, the folds gradually became smooth within 6 months, without the need for a second repair operation. If there was no improvement in the unsmooth folds at 6 months after surgery, we performed repair surgery using the traditional method and achieved a symmetrical and smooth double-eyelid line. There was no loss of the double-eyelid line and no hypertrophic scarring.

Regardless, our method still has some limitations. First, compared with the traditional method, the operation requires a slightly longer time, also being more complex and difficult for beginners. Second, the observation indices were subjective, while objective indices were not used. Objective indices will be used to further evaluate the surgical method in our subsequent studies.
